# Infection and innate immune mechanism of goose astrovirus

**DOI:** 10.3389/fmicb.2023.1121763

**Published:** 2023-01-26

**Authors:** Linhua Xu, Bowen Jiang, Yao Cheng, Yu He, Zhen Wu, Mingshu Wang, Renyong Jia, Dekang Zhu, Mafeng Liu, Xinxin Zhao, Qiao Yang, Ying Wu, Shaqiu Zhang, Juan Huang, Sai Mao, Xumin Ou, Qun Gao, Di Sun, Anchun Cheng, Shun Chen

**Affiliations:** ^1^Institute of Preventive Veterinary Medicine, Sichuan Agricultural University, Chengdu, Sichuan, China; ^2^Research Center of Avian Disease, College of Veterinary Medicine, Sichuan Agricultural University, Chengdu, Sichuan, China; ^3^Key Laboratory of Animal Disease and Human Health of Sichuan Province, Sichuan Agricultural University, Chengdu, Sichuan, China

**Keywords:** goose astrovirus, genome characteristics, homology, pathogenic mechanism, innate immune response

## Abstract

Goose astrovirus (GAstV, genus Avian Astrovirus, family Astrovirus) was first discovered in 2005, but was not considered as a pathogen of gosling gout until 2016. Since then, goose astrovirus has erupted in Chinese goslings, causing at most 50% of gosling deaths. By December 2022, the disease had become epidemic and prevailed in goose farms in Jiangsu, Shandong, Anhui, Henan, Guangdong, Liaoning, Sichuan and other places in China. The disease mainly affects goslings within 3 weeks old. The typical symptoms of goose astrovirus are large deposits of urate in the viscera, joint cavity and ureter surface of infected goslings. Goose astrovirus infection can trigger high levels of iNOS, limiting goose astrovirus replication. The ORF2 domain P2 of the goose astrovirus activates the OASL protein, limiting its replication. Goose astrovirus can also activate pattern recognition receptors (RIG-I, MDA-5, TLR-3), causing an increase in MHC-Ia, MHC-Ib and CD81 mRNA, activating humoral and cellular immunity, thereby hindering virus invasion. Goose astrovirus also regulates the activation of IFNs and other antiviral proteins (Mx1, IFITM3, and PKR) in the spleens and kidneys to inhibit viral replication. The innate immune response process in goslings also activates TGF-β, which may be closely related to the immune escape of goose astrovirus. Gaining insight into the infection and innate immune mechanism of goose astrovirus can help researchers study and prevent the severe disease in goslings better.

## Introduction

Goose astrovirus (GAstV, genus Avian Astrovirus, family Astrovirus) is a small, non-enveloped, single-stranded, positive-sense RNA avian astrovirus, which was first discovered in 2005 (Chen et al., 2021). The electron microscopy shows that the viral particles are spherical, about 28–30 nm in diameter (Li et al., 2022). Many members of the genus Avian Astrovirus, such as chicken astrovirus (CAstV), avian nephritis virus-1 (ANV-1), avian nephritis virus-2 (ANV-2), duck astrovirus (DAstV), duck astrovirus-1 (DAstV-1), Turkey astrovirus-1 (TAstV-1), Turkey astrovirus-2 (TAstV-2), all cause damage to liver, kidney and other organs or immune system (Zhu et al., 2022). Most of the clinical symptoms of astrovirus are severe diarrhea. In 2016, in the People’s Republic of China, goose astrovirus was first identified as a pathogenic factor causing gosling gout (He et al., 2022). Goose astrovirus has great genetic diversity and recombinant potential, demonstrating its ability to cause diseases in various hosts (Ji et al., 2020). Significantly, goose astrovirus can spread vertically from breeding geese to goslings, effectively infect Beijing ducks and muscovy ducks across the species barrier, increasing great threat to waterfowl industry development (Zhu and Sun, 2022). Although goose astrovirus caused serious economic losses, however, the understanding of the mechanism of goose astrovirus infection and immunization has not been fully revealed. Here, we summarized the current research progress of genomic characteristics, viral protein function, classification according to ICTV, pathological characteristics, diagnosis, epidemiology, homology, pathogenic mechanism, innate immune response and prevention vaccine development of goose astrovirus to study and prevent goose astrovirus effectively.

## Viral genome characteristics and viral protein function

**Figure 1:**
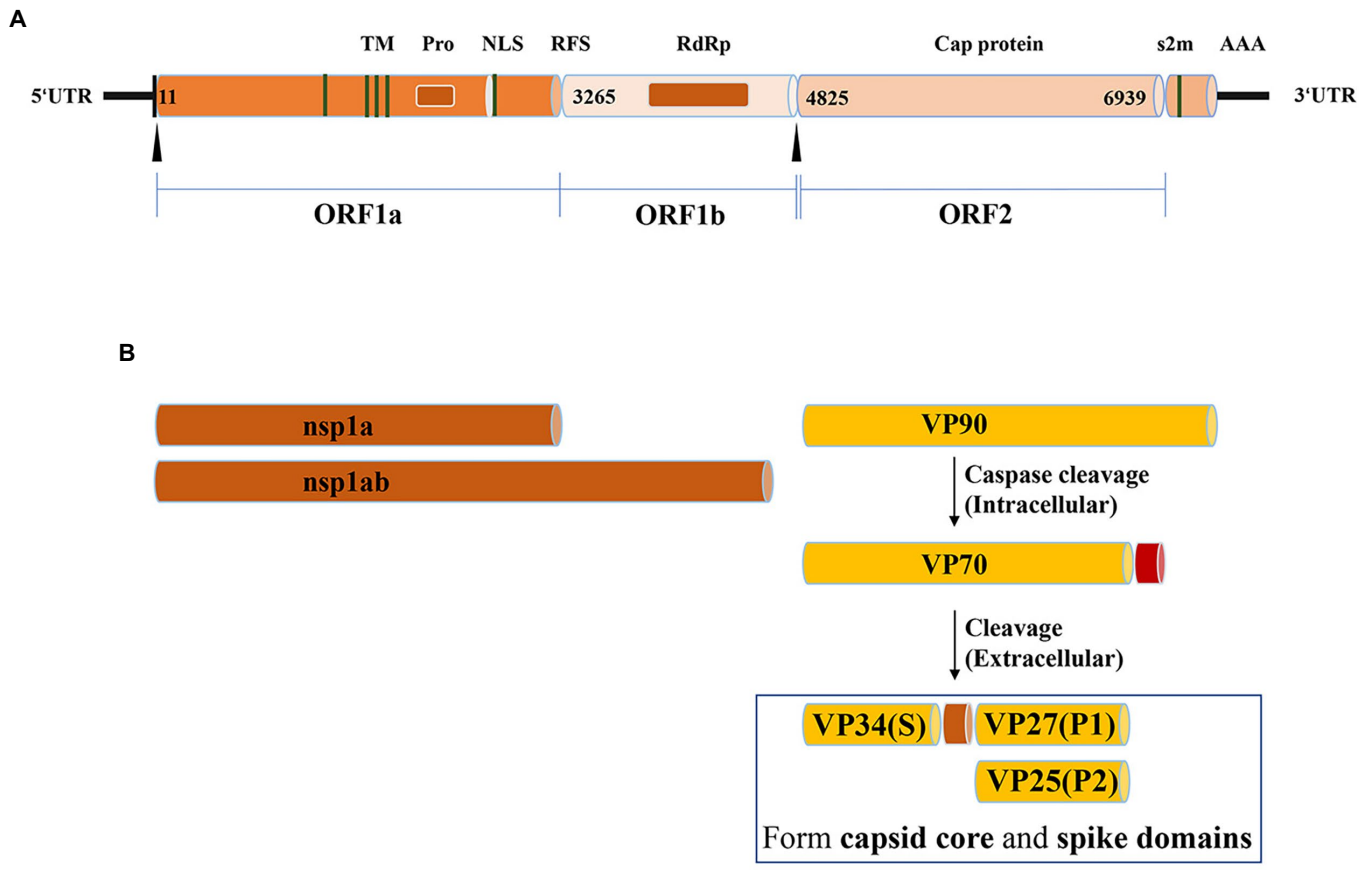
Summary of the genome characteristics. **(A)** The genome structure of goose astrovirus. **(B)** The protein structure of goose astrovirus.

Goose astrovirus is a single stranded sense RNA virus without envelope. Goose astrovirus is small and round. Transmission electron microscopy shows a star-like protrusion on its surface (Shen et al., 2022). The genome of goose astrovirus consists of a 5′-untranslated region (5′ -UTR), three open reading frames (ORF1a, ORF1b and ORF2), 3′-untranslated region (3’-UTR) and poly (A) Tail (Fu et al., 2022). The length of goose astrovirus genome varies by species. The length of goose astrovirus genome is about 7,200 bp, of which 5′-UTR and 3′-UTR are about 10 and 200 nt (Zhang X. et al., 2022). The overlapping areas of ORF1a and ORF1b include a conservative ribosomal frameshift (RFS) sequence and a downstream hairpin structure. RFS is essential to determine that RNA dependent RNA polymerase (RdRp) can be translated normally. The non-structural proteins of goose astrovirus are encoded by ORF1a and ORF1b, including transmembrane domain (TM), serine protease motif (Pro), nuclear localization signal (NLS) and RNA dependent RNA polymerase (RdRp) (Cui et al., 2022). The viral capsid protein encoded by ORF2 has significant diversity in the genome of goose astrovirus, which is composed of conservative region and variable region (Fei et al., 2022). The spike structure distributed on the outer surface of the virus particles is formed by a portion of the capsid protein encoded by the ORF2 region. Spike structures are structural barriers to goose astrovirus, which is involved in cell receptor recognition, host innate immune response and cytocytosis. The ORF1a and ORF1b encoded non-structural proteins of goose astrovirus mainly affect the replication and transcription of viral RNA. Capsid protein encoded by ORF2 is key to the response that stimulates innate immunity of goose astrovirus, and determines the intrusion and pathogenicity of goose astrovirus (Zhang F. et al., 2022; Figure 1A).

Goose astrovirus ORF2 genome encodes 704 amino acids. This region has no transmembrane region, no signal peptide sequence, and good local hydrophilicity (Li et al., 2021). Molecular analysis of the capsid protein (ORF2) genome region of goose astrovirus (GAstV) showed that it contained a neutralizing epitope (Wang et al., 2021). As observed in all astroviruses. Capsid proteins play a key role, so they are the key antigenic proteins on the surface of outer particles of astroviruses (Yin et al., 2021). The protrusions of the goose astrovirus are formed by the C-terminus of the ORF2-encoded capsid protein, capsid protein encoded by goose astrovirus is about 90kD (viral protein; VP: 90), which can be assembled into unformed virions in avian cells. (VP: 90) is digested and formed intracellularly by cysteine proteases (VP: 70), (VP:70) is digested extracellularly by trypsin as (VP:34), (VP:27) and (VP:25). (VP:34), (VP:27) and (VP:25) form gradually on the surface of the goose astrovirus. (VP: 34) is encoded by the S domain at the ORF2 N-terminal. (VP:27) and (VP:25) are encoded by ORF2 C-terminal domain P1 and domain P2 containing neutralizing antibody epitopes and susceptible cell receptor binding domains of goose astrovirus, respectively (Wang et al., 2021). (VP:34) and (VP:27) determine the infectivity of the virus, not (VP:25). (VP:34) forms the goose astrovirus core region structure. (VP:27) and (VP:25) together form goose astrovirus dimer structures, which contains many protrusions on the surface of goose astrovirus particles. Antigen cross reaction test shows that spike proteins play a role in receptor recognition, virus attachment and entry, and host immune response (Yin et al., 2021; Figure 1B).

## Classification of astrovirus according to ICTV

The International Committee on Taxonomy of Viruses (ICTV) divides astroviruses into two different generas according to their natural hosts, namely Mamastrovirus (MAstV) and Avastrovirus (AAstV) (Zhang M. et al., 2022). MAstV contains 19 internationally recognized astroviruses (MAstV 1–19) and 19 different astroviruses are mainly divided into two genotypes (GI/GII). Based on existing ICTV classification criteria, people classified 14 unclassified mammalian astroviruses into MAstV 20–33 (Wang Z. et al., 2022). P-distance will be helpful to the classification of goose astrovirus. Avian astrovirus classification considers the genetic standard based on ORF2 full length sequence. Goose astroviruses which p-distance of ORF2 complete sequence > 0.75 are classified as viruses of the same genotype (Zhang X. et al., 2022).

Three avian astrovirus genotypes (AAstV 1–3) have been specified internationally, and each genotype includes at least four different species at present. According to this standard, viruses with p-distance between 0.204 and 0.284 can be considered the same species. However, the *p*-distance of different viruses should be between 0.576 and 0.741.

Avian astroviruses can be further classified as avian astrovirus-1, avian astrovirus-2 and avian astrovirus-3. Turkey astrovirus-1 is a member of avian astrovirus-1, avian nephritis virus-1 and avian nephritis virus-2 make up avian astrovirus-2, Turkey astrovirus-2 and duck astrovirus-1 are important components of avian astrovirus-3. The full-length Cap gene is the genetic standard used by ICTV for further classification of avian astrovirus species. Capsid protein is the structural protein of astrovirus, responsible for the production of antibodies in the case of astrovirus infection_._ Two distant branches of goose astrovirus, goose astrovirus-1 and goose astrovirus-2, have been identified (Ren et al., 2022). By sequence alignment, the genome-wide similarity of goose astrovirus-1 and goose astrovirus-2 was 57.9%, the ORF2 genome similarity was 42.6% (Huang et al., 2021). Besides, the p-distance between goose astrovirus-1 and goose astrovirus-2 is 0.589–0.631, and the p-distance between different avian astroviruses is (0.657 ± 0.08), indicating that goose astrovirus-1 and goose astrovirus-2 can be regarded as different avian astroviruses (Wang et al., 2021). Significantly, since 2016, all the goose astrovirus strains in the goose astrovirus-2 branch have been related to the gout outbreak in geese. However, goose astrovirus under the goose astrovirus-1 classification is mainly characterized by gosling nephritis (Ding et al., 2021). According to the phylogenetic relationship of Cap gene, goose astrovirus-2 with Turkey astrovirus-2 and duck astrovirus-2 can be classified into avian astrovirus-3, while goose astrovirus-1 with Turkey astrovirus-1 can be classified into avian astrovirus-1. Similar to the classification of MAstV, ICTV classifies four other unclassified avian astroviruses as avian astrovirus 4–7, avian astrovirus-4 is divided into chicken astrovirus, pigeon nephritis virus is an important component of avian astrovirus-5. Avian astrovirus-6 contains pigeon astrovirus, avian astrovirus-7 is classified into wild pigeon astrovirus (An et al., 2020).

## Pathological characteristics of goose astrovirus

The way of isolating goose astrovirus is to amplify and isolate the virus on avian embryo or avian cell lines in most reports. Some reports showed that the primary goose embryonic kidney cells (GEK) could also achieve virus proliferation. Other primary cells, such as goose embryonic hepatocyte (GEH), and goose embryonic fibroblasts (GEF), have also been used to isolate goose astrovirus in some studies (Dalbeth et al., 2021). Goose embryonic kidney cells (GEK) have the best virus growth among them (Zhang et al., 2018). The replication ability of goose astrovirus in avian embryos was initially poor, but in some specific embryos, the best replicated goose astrovirus was 100% fatal to the embryo. Since there is no specific pathogen free (SPF) and easily isolated avian cell models, other pathogens must be excluded from avian embryos every time the virus spreads. This makes the spread of the virus very inconvenient (Zhao et al., 2022). Many studies have also used SPF chicken embryo or duck embryo instead of goose embryo to reproduce goose astrovirus. Goose astrovirus can reproduce in SPF chicken embryos through the allantoic cavity pathway or with the help of trypsin in some cell lines, and some strains fail to adapt to chicken embryo and duck embryo. Although goose astrovirus can replicate in avian cell lines, some strains of goose astroviruses can cause lesions in specific avian cells, while others do not. There is no clear and reliable isolation and transmission of goose astroviruses, which makes it particularly difficult to further study the molecular mechanisms and prevention of goose astroviruses. In addition, goose astrovirus can be mixed with a variety of viruses, especially avian influenza virus, goose parvovirus, tembusu virus, and so on. Although single goose astrovirus infection still accounts for the vast majority of sick geese, multiple infections make isolation of goose astrovirus particularly difficult (Liao et al., 2015).

Due to their high genetic diversity and recombination potential, astroviruses have the ability to cause a wide range of diseases in a variety of host species, so many animals, including humans, mammals and poultry, may be infected with astroviruses (Bukkitgar et al., 2021; Gutierrez et al., 2022). Pathogens, especially avian astrovirus infections, are considered one of the most critical causes of large-scale farming of goslings gout. More and more studies have shown that several astroviruses, including ANV-1, chicken astrovirus and goose astrovirus, are related to the occurrence and development of avian gout (He et al., 2020). China has reported a new goose astrovirus (GAstV) in 2016 which resulted in the death of 2–20% of the young geese aged 4–16 days, and restricted the development of Chinese goose industry (Ren et al., 2020). Goose astrovirus can be replicated in many organs which indicates that goose astrovirus has certain tissue tropism and targeting organ (Ren et al., 2020). The number of goose astrovirus copies in the goslings’ kidneys after infection by goose astrovirus is significantly higher than in other tissues. These results suggest that the kidneys may be the target organs for goose astrovirus infection (Liu et al., 2022). In addition, the number of viral copies of the spleen and liver is also very high. Some studies have shown that eosin staining shows hemorrhage of intermediate substance in kidney slices, necrosis of renal tubules and swelling of glomeruli, and uric acid crystallization and vacuolar degeneration of liver cells in liver slices autopsy revealed severe urate deposition in the viscera and joints (Liu M. et al., 2019).

## Diagnosis, epidemiology, homology of goose astrovirus

There is an urgent need for new high-precision diagnostic tools with sensitivity, specificity and high throughput for the diagnosis and monitoring of goose astrovirus. In this case, timely identification of goose astrovirus will be very beneficial. It has been reported that the use of dual TaqMan real-time RT-qPCR can be used to identify the two kinds of goose astrovirus, which Supplemented with the blank of diagnostic tools (Yin et al., 2020). For the reason that monoclonal antibodies can also detect VP27 proteins in goose astrovirus-infected tissues, the newly prepared anti-VP27 monoclonal antibody 2AF 11 may become an efficient tool to act as a special diagnostic material for goose astrovirus; such as colloidal gold immunochromatography, double antibody sandwich ELISA and ICS can all become good choices for diagnosing goose astrovirus. The sandwich ELISA and indirect immunofluorescent antibody technique (IFA) methods established on this basis provide an efficient and rapid serodiagnostic tool for the diagnosis and differentiation of goose astrovirus-1 and goose astrovirus-2 (Wang A. et al., 2022). Indirect ELISA is very sensitive, which is four times that of Western blotting. It can also be used to measure the antibody level of astrovirus. Recently, a colloidal gold immunochromatographic assay based on monoclonal antibodies was developed for rapid diagnosis of goose astrovirus. Some reports also point out LAMP and ICS, which are designed to diagnose goose astrovirus, also contribute greatly to diagnose disease rapidly. EvaGreen qLAMP is characterized by rapid, simple, sensitive and specific analysis, and it can be performed without a thermal circulator. Compared with SYBR Green based LAMP analysis, EvaGreen based LAMP analysis overcomes these shortcomings. The qLAMP analysis showed high sensitivity and no cross reaction with other viruses. Rapid diagnosis depends on RT-LAMP analysis. The sensitivity of this method is equivalent to that of PCR (Yuan et al., 2018, 2019). ICS has better accuracy, reliability and clinical applicability in rapid diagnosis of target viruses (Yuan et al., 2018). The results obtained from tissue homogenate supernatant samples are low, which may be due to the influence of polyclonal antibodies against goose astrovirus. ICS can be stored at 4°C for 6 months; Therefore, ICS has the best clinical practicability. It has reported that a new diagnostic immunochromatographic strip (ICS) method can be used to diagnose goose astrovirus. The reproducibility of the test was confirmed in the tests within and between sensitivity, and the variation range was 0.61–2.21%. In addition, this method is convenient and requires very simple steps and very little time to quickly complete the diagnosis of goose astrovirus. Comparing with the previous ICS test, this method does not require destruction of the cell membrane, but its specificity and stability are not significantly reduced. Besides, methods of mass spectrometry analysis were also reported to diagnose 10 goose viruses at one time, including goose astrovirus. By optimizing experimental conditions, the multiple PCR and mass spectrometry methods established in the laboratory can detect all kinds of viruses accurately without error, indicating that the combination of mass spectrometry and PCR methods has good accuracy (Dai et al., 2022). All molecular biology and serology tools are fast, efficient, sensitive and specific, making them indispensable for in-depth study of the pathogenic mechanism and innate immune response of goose astrovirus.

When goose astrovirus was first proved in geese showing disease in 2016, gout disease of goslings caused by unknown causes was found in coastal areas of Southeast China (Xi et al., 2022). In the same year, the disease rapidly spread to most goose producing areas in China, including many coastal provinces and neighboring areas, including Jiangsu, Zhejiang, Fujian, Shandong, Liaoning, Heilongjiang, Anhui, Jiangxi, Hebei, Henan, Hunan, and other 15 provinces, where goose astrovirus has been reported. Subsequently, the number of cases reported in South China and Southwest China continued to increase, including Guangdong Province in 2018 and Sichuan Province in 2020. The external manifestation of the infected goslings is depression, loss of appetite, and some goslings have grey eyelids and reduced weight. Infected by goose astrovirus, the mortality rate of goslings reaches 10–20%, and the maximum is 50%. Histopathological changes were typical, including glomerular swelling, necrosis and renal tubular epithelial cell abscission. At autopsy, severe urate deposition will be found (Ma et al., 2021).

**Figure 2:**
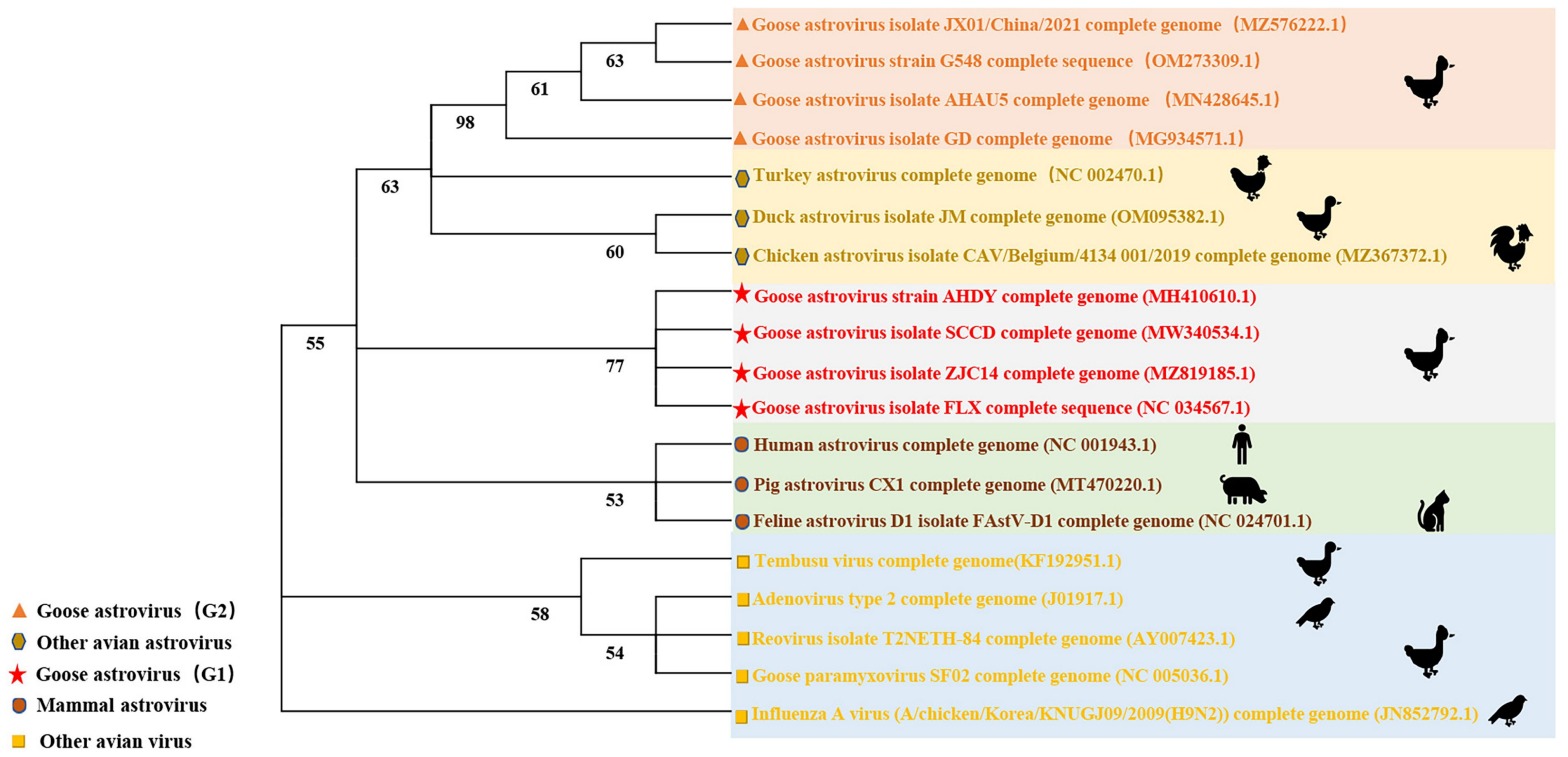
Phylogenetic analysis of goose astrovirus. Phylogenetic trees were generated based on whole genome sequence using the neighbor-joining method with 1,000 bootstrap replicates as determined with MEGA 7.0 software.

ICTV identified three kinds of avian astrovirus: avian astrovirus-1 (AAstV-1), avian astrovirus-2 (AAstV-2), and avian astrovirus-3 (AAstV-3) in 2011. Mammalian astroviruses are classified into mammalian astroviruses 1–19 and mammalian astroviruses 20–33. Avian astrovirus is divided into avian astroviruses 1–7, avian astrovirus-1 is divided into goose astrovirus-1 and Turkey astrovirus-1, avian astrovirus-2 is divided into avian nephritis virus-1 and avian nephritis virus-2, avian astrovirus-3 is divided into Turkey astrovirus-2, duck astrovirus-1, goose astrovirus-2 and duck astrovirus-2 (An et al., 2020). In this review, 10 goose astroviruses, 3 other avian astroviruses, 3 mammalian astroviruses and 5 other avian viruses were selected for phylogenetic analysis. The phylogenetic tree of the full-length gene sequence shows that most same avian astrovirus species are closely related, while the goose astrovirus strain is classified as goose astrovirus-1 and goose astrovirus-2, which are related but have different pathological characteristics. Analysis of genome-wide sequence homology of goose astroviruses reveals that goose astrovirus-2 is similar to AAstV-3 homology, while goose astrovirus-1 is similar to AAstV-1 homology. Recent reports show that all gout related goose astrovirus strains are clustered in goose astrovirus-2 group but goose astrovirus-1 is closely related to gosling enteritis. More in-depth sequence alignment analysis is important for further explaining the pathogenicity of different goose astroviruses (Wan et al., 2018). Astroviruses with ORF2 a sequence homology > 75% were thought as the same kinds of astroviruses. However, further sequence analysis showed significant wide disparities between goose astrovirus-1 and goose astrovirus-2 which may be the reason for the different pathological phenomena between goose astrovirus-1 and goose astrovirus-2 (Figure 2).

## Pathogenic mechanism of goose astrovirus

**Figure 3:**
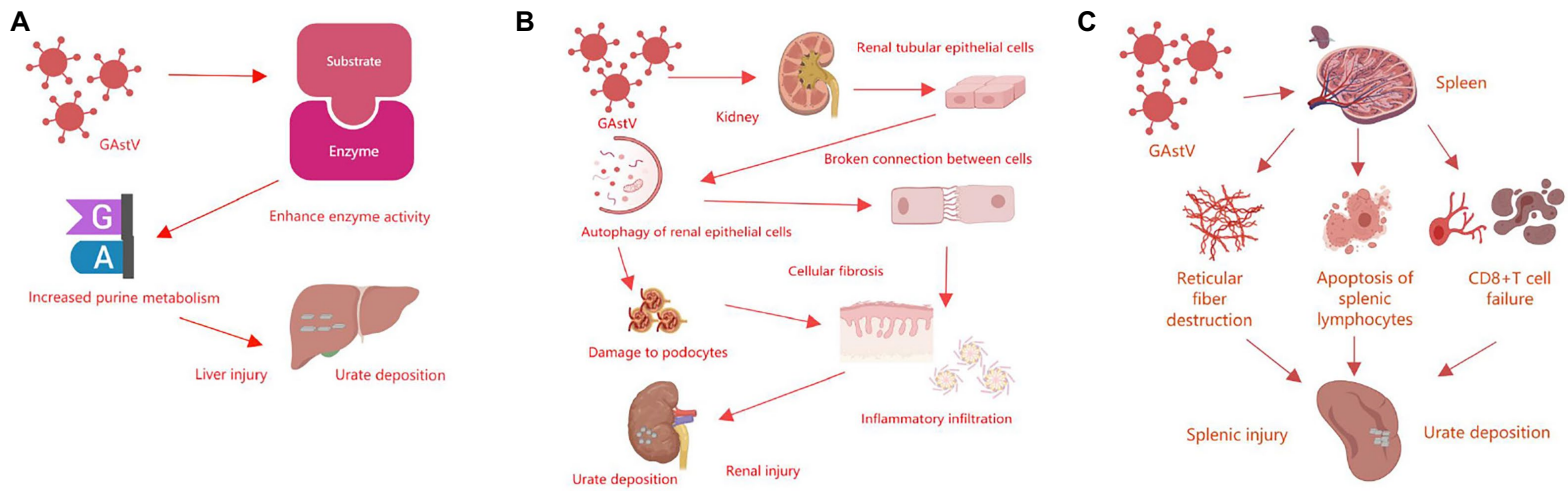
Summary of the pathogenic mechanisms of goose astrovirus. **(A)** Pathogenic mechanisms of goose astrovirus in liver. **(B)** Pathogenic mechanisms of goose astrovirus in kidney. **(C)** Pathogenic mechanisms of goose astrovirus in spleen.

Gosling gout is a typical feature of goose astrovirus infection. Studies have shown that the activities of xanthine oxidase (XOD) and adenosine deaminase (ADA), as well as amount of mRNA of xanthine deaminase, adenosine deaminase, ribophosphate pyrophosphate aminotransferase and ribophosphate thermophosphate synthetase increase, while the mRNA levels of renal drug resistance related proteins and the activities of Na-K-ATPase decrease (Yugo et al., 2016; de Souza et al., 2019). These results indicate that goose astrovirus infection can lead to goslings’ livers damage, then promote the formation of uric acid and enhances expression activity related enzymes in the liver, increase purine metabolism of the liver, and lead to urate deposition in blood and severe gout in goslings (Wei et al., 2020; Wu et al., 2020; Yang et al., 2020; Figure 3A). Goose astrovirus can cause significant changes in permeability of renal epithelial cells, while the increase in the permeability of renal epithelial cells may lead to gout (Yu et al., 2020). Infectious factors include infectious bronchitis virus of renal, inclusion bodies caused by avian adenovirus inflammation and oviposition syndrome76 (EDS-76), cryptosporidiosis of kidney, and goose astrovirus. For example, chicken kidney pathogenic infectious bronchitis virus (IBV), which can destroy renal tissue and thus deactivate renal cells (Liu M. et al., 2019). Goose astrovirus infection leads to autophagy of renal epithelial cells, destruction of the boundaries and intercellular junctions of renal tubular epithelial cells, podocyte damage, increased fibrosis and inflammatory cell infiltration, thus leading to kidney damage of infected goslings (Chen et al., 2020; Yin et al., 2020; Chen et al., 2021; Figure 3B). These results allow us to understand more deeply the pathogenesis of goose astrovirus. Goose astrovirus contains three ORFs. Based on conserved genomic elements, some researchers hypothesized that they share a common replication mechanism. IFNs are generated after PRR activation. PRR plays a key role in virus inhibition by inducing multiple IFNs stimulating genes, and the products of these genes have direct antiviral activity. The increase of antiviral protein production may help to inhibit virus invasion (Liu N. et al., 2019; Wan et al., 2019). Some investigations have shown that goose astrovirus infection is an innate immune response of the host (Yuan et al., 2018; Xi et al., 2020a). Goose astroviruses are at present in large numbers in lymphocytes and macrophages in the spleen and can observe large amounts of cell necrosis, suggesting that goose astroviruses can infect the spleen and inactivate its immune cells (Yuan et al., 2019). Goose astrovirus can also induce apoptosis of splenic lymphocytes, destruction of reticular fibers and depletion of CD8 + T cells (Zhang et al., 2018; Figure 3C). These survey studies indicate that hosts of goose astrovirus may have crossover, which may be an important cause of goose astrovirus infection with other waterfowl (Jin et al., 2018).

Urate deposits are major nitrogen-containing wastes excreted by poultry. Deposition of urate is caused by the presence of hyperuricemia complications in the body by sodium urate crystals. Uric acid salt is the main nitrogenous waste in poultry (Yang et al., 2018). The results showed that the serum uric acid level of goslings infected by goose astrovirus was significantly higher than that of postnatal goslings, indicating that goose astrovirus may lead to disrupt liver function and increase purine levels (Niu et al., 2018). This prediction is consistent with experimental measurements of viral load changes, and pathological changes can be observed in the corresponding tissues (Zhang et al., 2018). The liver is the organ with the second highest number of copies of viruses than kidneys in the goose detection organ, and the organ has significant urate deposits (Liu et al., 2018). Uric acid is produced in the final stages of urine excretion. Uric acid production involves a variety of enzyme classes. These studies have shown that goose astrovirus can enhance uric acid production levels by enhancing the activity of cyanuric acid-generating enzymes (Zhang et al., 2018). Similarly, nephrogenic infectious bronchitis virus infection in chicks causes kidney gout by increasing the transcription of xanthine oxidase mRNA in the kidneys of broilers. The formation of hyperuricemia is not only related to the deposition of urate, but the metabolism of kidneys is also important to the formation of hyperuricemia. In this study, the serum creatinine level did not change after 7 and 14 dpi of goose astrovirus infection, indicating that goose astrovirus infection may not affect glomerular function. This is similar to the process of the kidney following infection. No glomerular injury was found, but tubular injury was observed after infection with goose astrovirus. Degenerative necrosis of renal epithelial cells and inflammatory cells can also be found during pathologic section observation (Zhang et al., 2017).

Besides, kidney as the organ with the highest number of copies of the virus after goose astrovirus infection, the secretion and reabsorption of the renal tubules occur at the proximal end of the tubules. Uric acid transporter 1 is an important factor affecting the reabsorption of uric acid (Yugo et al., 2016); however, there is no similar sequence in the poultry system. At present, MRP4 and oat protein are closely related to uric acid transport in poultry (Yi et al., 2022). Multidrug resistance associated protein 4 is located in the apical membrane of proximal renal epithelium and is considered as the main apical outlet of uric acid from cells to lumen (Yang et al., 2021). Organic anion transporter 1 and OAT3 produce sodium gradients through Na-K-ATPase and are responsible for the transport of urate across the basolateral membrane (Boocock et al., 2020). In other studies, the MRP4 mRNA level and Na-K-ATPase activity of infected goslings were particularly lower than those of the control group, showing that goose astrovirus infection reduced kidney secretory function (Wang H. et al., 2022). Unfortunately, there are no reports on goose mRNA sequences; therefore, the change of OAT expression or activity after goose astrovirus infection remains to be studied.

## The innate response to goose astrovirus

Goose astrovirus caused damage to the immune system of the host. INOS participates in most processes of innate immune response and plays an irreplaceable role in the protection against virus infection (Buiret et al., 2022). Expression of iNOS was found to be significantly upward controlled by goose astrovirus infection. Strong production of iNOS can make the host immune to viral infection. Studies have shown that goose astrovirus infection induces the production of iNOS, which restricts the replication of astroviruses (Bianchi et al., 2018). Previous reports also indicate that interferon-I systems limit the replication of human astroviruses and provide protection of human astrovirus by changing barrier permeability (Bidin et al., 2012; Boehm et al., 2019). In some studies, ORF2 actually activated innate immune responses and induced elevated levels of 2′-5’oligoadenylate synthetase-like (OASL) *in vivo* and *in vitro*. It should be noted that goose astrovirus triggers OASL by ORF2 to restrict its replication. The truncation analysis also showed that the domain P2 of ORF2 facilitate the stimulation of OASL, while the acid C terminal of ORF2 weakened activation (Ren et al., 2020). Capsid protein of goose astrovirus encapsulates viral nucleic acid, determines cell orientation, mediates virus adsorption, and stimulates host immune response to goose astrovirus. However, there are comparatively few studies on capsid protein of goose astrovirus. With the continuous discovery of goose astrovirus-antigen epitopes, the understanding of goose astrovirus-cap’s function will be further developed.

Besides activating iNOS and OASL, goose astrovirus infection can induce activation of pattern recognition receptors, including RIG-I like receptor (RIG-I) gene, Melanoma Differentiation-Associated Gene 5 (MDA-5), Toll-like receptor 3 (TLR-3). Pattern recognition receptors (PRRs), including RIG-I/MDA5 and TLR3, increased MHC-Ia, MHC-Ib and CD81 mRNA levels in patients with goose astrovirus infection, indicating that humoral and cellular immune responses have been activated (Dalbeth et al., 2021). Goose astrovirus infection also induces activation of key adaptive molecules (MyD88, MAVS and IRF7), and regulates IFN-α in spleen and kidney and other antiviral proteins (Mx1, IFITM3 and PKR). Pattern Recognition Receptors (PRRs), including RIG-I, MDA5 and TLR3, participate in the immune response of the host of the goose astrovirus and PRRs can activate the goose astrovirus IFN-β, IFN-β-induced IFN (such as IFN-I), inflammatory cytokines (such as IL-8, IL-10 and TNF-β) in the kidneys and other antiviral proteins (such as Mx1, IFITM3, PKR), which have been reported before. The enhanced expression of TLR3, RIG-I and MDA-5 mRNA in the spleen and kidney caused by goose astrovirus infection indicates that the innate immune system is activated, which can help suppress viral invasion. These results were confirmed by the up regulation of key linkers (MyD88 and IRF7). The relative content of TLR3 and OASL is significantly higher than 14 dpi at 7 dpi, which may be due to negative feedback regulation and decreasing virus copies. In addition, CD81 and MHC-Ia have reduced mRNA levels at 14 dpi compared to 7dpi. Whereas CD81 T cells are essential for the maturation of B cells and the specificity of antibodies, goose astrovirus may not cause a strong immune response (Meyer et al., 2020). All results are important reasons for the lack of significant increase in MHC-Ia relative content at 7dpi (Beressa et al., 2021).

**Figure 4:**
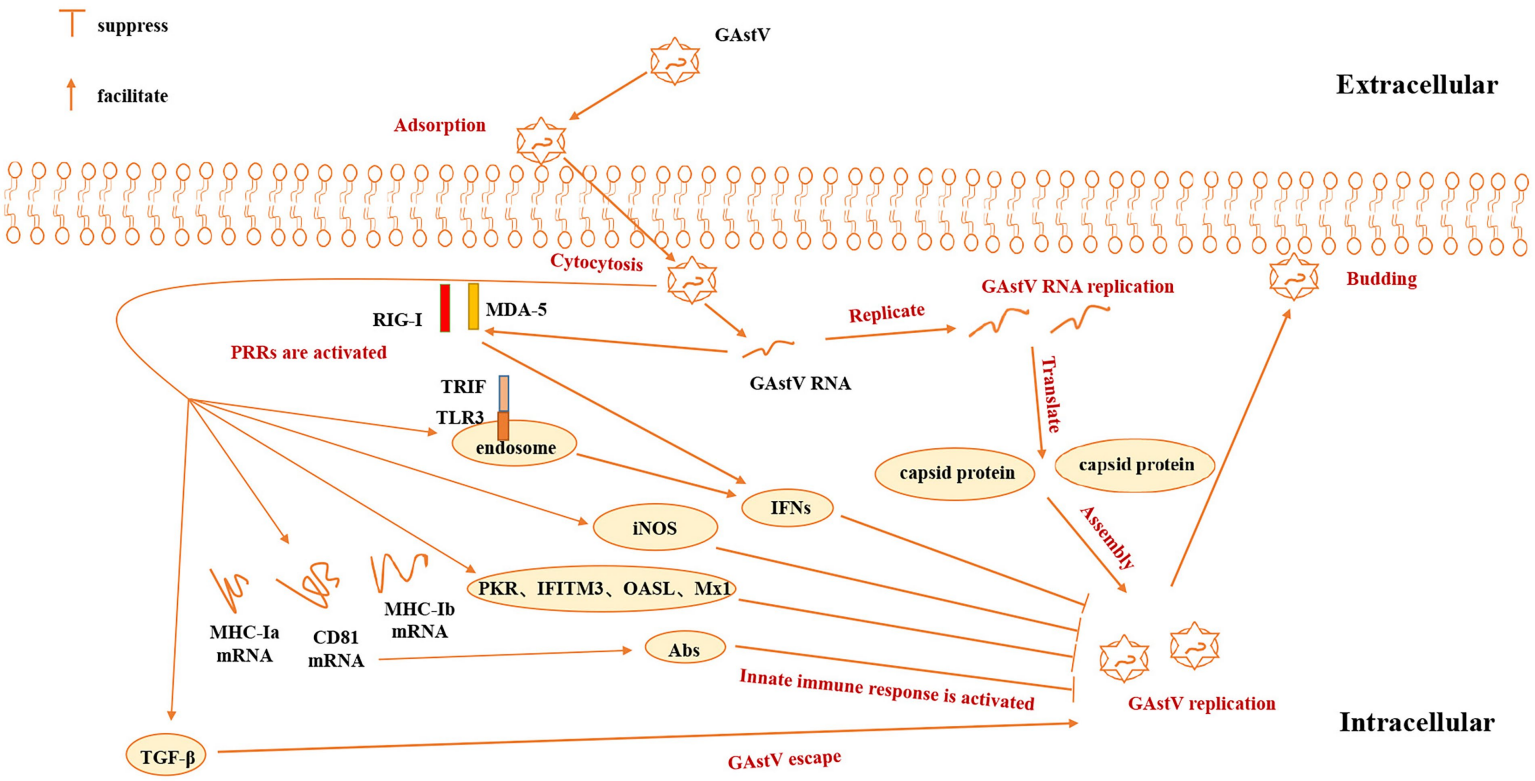
Innate immune mechanism of goose astrovirus. GAstV, goose astrovirus; PRRs, pattern recognition receptors; PKR, IFITM3, OASL, Mx1, antiviral proteins; RIG-I, MDA-5, TLR-3; pattern recognition receptors; Abs, antibodies; TGF-β, transforming growth factor-β; iNOS, inducible nitric oxide synthase.

Goose astrovirus can also activate innate response in other approachs. Recently, overexpression of cytokines in cells infected with goose astrovirus is also reported (Wu et al., 2021). The major molecules that connect the spleen and kidneys, including Myeloid Differentiation factor 88, the mitochondrial antiviral signaling gene, and interferon 7 regulation factor also make major contributions in the innate immunity of goose astrovirus. Expression of the transmembrane protein-mediated antimyxovirus 1, IFN-α is also related to goose astrovirus. Goose astrovirus infection also improves expression of spleen interferon-γ (IFN-γ) and interferon activated transmembrane protein 3 of metabolic organs. In addition, relative amounts of induced nitric oxide synthase in the spleen and kidneys is very high and the expression of interleukin-1 (IL-1) and IL-8 in the spleen is upregulated after being infected by goose astrovirus (Bidin et al., 2012). A high level of virus copies has been observed in renal epithelial cells, which may be due to kidney lesions and a disorder of uric acid excretion caused by the goose astrovirus. However, there are reports of varying degrees of decrease in relative levels of pro-inflammatory factor IF-6 after 3 days. At the same time, the relative content of IL-6 mRNA and IL-6 mRNA in the kidneys decreased to some extent. Levels of TGF-β have been reported to be significantly elevated in the spleens and kidneys, which may be closely related to immune escape from goose astrovirus (Beressa et al., 2021; Figure 4). All in all, there are more in-depth innate immune response mechanism that we need to study.

## Prevention vaccine development of goose astrovirus

Goose astrovirus is a very special waterfowl virus, and its unique pathological characteristics are important reasons why it is long ignored. Even over the past decade, some believe that the reason for gosling gout is high protein intake. With the advancement of technology and the deepening of research, goose astrovirus is considered as the pathogen of the gosling gout (Xi et al., 2020b). However, veterinary treatment of goose astrovirus is limited to the elimination of gout symptoms, the prevention strategy mainly comes from the timely diagnosis of goose astrovirus, goose astrovirus can cause host metabolism disorders, leading to symptoms of gosling gout. Metabolic therapy can relieve symptoms of gout, but does not make it difficult for goose astrovirus to replicate in the goslings. We need to think about the whole process of infecting our hosts and understand the key links in preventing and treating goose astrovirus in order to better design a vaccine that efficiently eliminates goose astrovirus.

Henceforth, a deeper exploration of the pathogenic and innate immune response mechanisms of goose astrovirus may fundamentally address the gosings gout caused by goose astrovirus (Xu et al., 2019). The development of DNA recombination and reverse genetics over the past few decades has dramatically changed the pattern of vaccine development. In addition, advanced transcriptomes, proteomes, and research in polygenomics provides a great deal of key information for finding reliable protective antigens.

With the continuous innovation and progress of science and technology, DNA carrier vaccines and nano vaccine preparations using liposomes as carriers have developed continuously, but because of the long research and development cycle, the cost is difficult to control and the mass production is difficult to apply to the preparation of avian vaccine. With a better understanding of the waterfowl immune system, studies have developed avian adenovirus as a vaccine vector, and vaccines against goose astrovirus and adenovirus have had a better impact in the laboratory phase. Goose astrovirus (GAstV) and goose-borne Neustadt virus (NDV) are both widespread sources of infection in goslings (Xu et al., 2019). Molecular analysis of the genomic region of goose astrovirus (GAstV) capsid protein (ORF2) showed that it contained a neutralized epitope. Higher protein content in waterfowl feed and infection with a variety of avian diseases can cause similar pathological symptoms of goose astrovirus. Timely detection and diagnosis of goose astrovirus is still the only widely used means. The efficient isolation of different strains of goose astroviruses can further provide valuable resources for host interaction, molecular biology research and the development of further diagnostic therapeutic methods (Ren et al., 2020).

To date, no vaccine or other measure has been able to adequately and effectively control and prevent the outbreak and epidemic of goose astrovirus. Although the stability of goose astroviruses in various environments was not evaluated. However, other kinds of astroviruses have good adaptability in various environments. Not only can it resist the change of disinfectant concentration, temperature, acid-alkalinity, salt-ion concentration, but it also has some viability for more complex environment. Strict disinfection and prevention can significantly reduce the likelihood of infection with goose astrovirus. Because different species of goose astrovirus may be mixed with different other avian viruses, studying a proven method to eliminate goose astrovirus is essential for the development of the poultry industry. Capsid proteins can trigger neutralizing antibodies in goose astrovirus (Zhang et al., 2019). The latest study involved the insertion of the goose astrovirus cap gene into a weakened NDV strain to prepare a divalent vaccine. Although the vaccine activates antibodies to goose astrovirus and NDV, it may cause pathogenic goose astrovirus and NDV to replicate in the goslings. The rNDV/goose astrovirus-cap virus could become a safe, stable and effective bivalent vaccine. Recombinant goose source Neustadt virus vector goose astrovirus vaccine can not only prevent Neustadt virus but also goose astrovirus (Xu et al., 2019). However, it has not been widely used. At the laboratory stage, there are reports of traditional vaccines such as the goose astrovirus attenuated live vaccine (Zhang et al., 2018), goose astrovirus gene recombination subunit vaccines and inactivated goose astrovirus vaccines are being developed. However, there is currently no commercial vaccine.

## Conclusion

High or low levels of critical feed, especially trace elements, in waterfowl feed can lead to goslings gout before attention begins to be paid to goose astrovirus. Eating fat in large quantities can also cause the goslings, ground metabolism to be dysregulated, thereby increasing blood uric acid levels and causing gout. Poor farming environments (such as dark, high-density farming, wet feeding sites) can cause gout in goslings. Therefore, maintaining a good feeding environment is key to reducing morbidity. In addition to the aforementioned factors, goose astrovirus is now considered to be a key cause of gosling gout.

Goose astrovirus can mainly cause renal epithelial cell autophagy, renal tubular epithelial cell boundary and inter-cell junction destruction, foot cell damage, increased fibrosis and inflammatory cell infiltration through infection, resulting in damage to the kidneys of infected chicks and the production of urate deposits in the kidneys. Goose astrovirus infection can induce apoptosis of spleen lymphocytes, destruction of reticular fibers and depletion of CD8 + T cells. Goose astrovirus infection can also promote purine metabolism by enhancing the activity of uric acid-producing related enzymes, causing urate deposition in the liver. The potential cross-species and vertical transmission of the goose astrovirus and the lack of vaccines pose great challenges for disease control and prevention. Many astroviruses (ANV-1, CAstV, GAstV) have been shown to be relevant with avian influenza occurrence and prevalence. Genetic recombination of viruses is a key reason why viruses are able to evolve continuously. Single-stranded RNA viruses are more prone to recombination than double-stranded DNA viruses because of their single-stranded genome structure. There have been some reports of multiple recombinations of human astroviruses during epidemics, suggesting that goose astroviruses may also be more prone to recombination, resulting in more different species of goose astroviruses, which may have different pathogenic characteristics and different genomic structures (Ren et al., 2020). There have been few previous reports of goose astrovirus restructuring (Zhang et al., 2018; Zhang F. et al., 2022). In further infection experiments, it is necessary to test the potential for transmission of the strain across species and its pathogenicity to brood geese. The innate immune response mechanism of goose astrovirus mainly includes that infection of goose astrovirus can trigger high levels of iNOS and thus limit replication of goose astrovirus (Buiret et al., 2022). The ORF2 P2 domain of goose astrovirus can activate the OASL protein and thus limit its replication (Ren et al., 2020); goose astrovirus can also activate pattern recognition receptors (RIG-I, MDA-5, TLR-3), leading to an increase in MHC-Ia, MHC-Ib and CD81 mRNA, activating humoral and cellular immunity, and thus hindering virus invasion. This can be demonstrated by an increase in key adaptive molecules (MyD88, MAVS, and IRF7). Goose astrovirus also modulates IFNs and activation of other antiviral proteins (Mx1, IFITM3, and PKR) in the spleen and kidneys to inhibit virus replication (Dalbeth et al., 2021). The innate immune response process in geese also activates TGF-β, which may be closely related to the immune escape of goose astrovirus (Beressa et al., 2021).

In a conclusion, research on the pathogenic mechanism and innate immune response of goose astrovirus are limited at present. With the continuous discovery of the epitope of capsid protein antigen of goose astrovirus and the continuous discovery of cell-specific receptors, the understanding of the infection and immune mechanism of goose astrovirus will be more and more thorough, there is still a long way to go to study and prevent goose astrovirus.

## Author contributions

LX, BJ, YC, YH, ZW, MW, RJ, DZ, ML, XZ, QY, YW, SZ, JH, SM, XO, QG, DS, AC, and SC: conceptualization. LX and SC: data curation. BJ: formal analysis. AC and SC: funding acquisition and project administration. SC: resources and supervision. LX: writing–original draft and review and editing. All authors contributed to the article and approved the final manuscript.

## Funding

This work was funded by grants from the National Key Research and Development Program of China (2022YFD1801900), National Natural Science Foundation of China (32272976), Sichuan Provincial Department of science and technology international scientific and technological innovation cooperation (2022YFH0026), the earmarked fund for China Agriculture Research System (CARS-42-17), and the Program Sichuan Veterinary Medicine and Drug Innovation Group of China Agricultural Research System (SCCXTD-2021-18).

## Conflict of interest

The authors declare that the research was conducted in the absence of any commercial or financial relationships that could be construed as a potential conflict of interest.

The reviewer ZX declared a shared affiliation with the authors to the handling editor at the time of review.

## Publisher’s note

All claims expressed in this article are solely those of the authors and do not necessarily represent those of their affiliated organizations, or those of the publisher, the editors and the reviewers. Any product that may be evaluated in this article, or claim that may be made by its manufacturer, is not guaranteed or endorsed by the publisher.
